# Lightning Injury is a disaster in Bangladesh? - Exploring its magnitude and public health needs

**DOI:** 10.12688/f1000research.9537.1

**Published:** 2016-12-29

**Authors:** Animesh Biswas, Koustuv Dalal, Jahangir Hossain, Kamran Ul Baset, Fazlur Rahman, Saidur Rahman Mashreky

**Affiliations:** 1 Centre for Injury Prevention and Research, Bangladesh (CIPRB), Dhaka, Bangladesh; 2 School of Health Sciences, Örebro University, Örebro, Sweden

**Keywords:** Lightning injury, incidence, disaster, Bangladesh

## Abstract

**Background:** Lightning injury is a global public health issue. Low and middle-income countries in the tropical and subtropical regions of the world are most affected by lightning. Bangladesh is one of the countries at particular risk, with a high number of devastating lightning injuries in the past years, causing high mortality and morbidity. The exact magnitude of the problem is still unknown and therefore this study investigates the epidemiology of lightning injuries in Bangladesh, using a national representative sample.

**Methods:** A mixed method was used. The study is based on results from a nationwide cross-sectional survey performed in 2003 in twelve randomly selected districts. In the survey, a total of 819,429 respondents from 171,336 households were interviewed using face-to-face interviews. In addition, qualitative information was obtained by reviewing national and international newspaper reports of lightning injuries sustained in Bangladesh between 13 and 15 May 2016.

**Results: **The annual mortality rate was 3.661 (95% CI 0.9313–9.964) per 1,000,000 people. The overall incidence of lightning injury was 19.89/100,000 people. Among the victims, 60.12% (n=98) were males and 39.87% (n=65) were females. Males were particularly vulnerable, with a 1.46 times increased risk compared with females (RR 1.46, 95% CI 1.06–1.99). Rural populations were more vulnerable, with a 8.73 times higher risk, than urban populations (RR 8.73, 95% CI 5.13–14.86). About 43% of injuries occurred between 12 noon and 6 pm. The newspapers reported 81 deaths during 2 days of electric storms in 2016. Lightning has been declared a natural disaster in Bangladesh.

**Conclusions:** The current study indicates that lightning injuries are a public health problem in Bangladesh. The study recommends further investigations to develop interventions to reduce lightning injuries, mortality and related burden in Bangladesh.

## Introduction

Lightning injury is a global public health problem representing the leading cause of weather-related death after tornadoes, flash floods and hurricanes. The incidence rates of lightning injury are probably higher than registered since there is no referral and information centre where data are collected and stored ^1^. Lightning strikes the earth more than 100 times each second, totalling 8 million times every day. An estimated 50,000 thunderstorms occur each day, causing fires and injuries ^2^. Worldwide, mortality from lightning is estimated at between 0.2 and 1.7 deaths/1,000,000 people, affecting mainly the young and people who work outdoors ^3, 4^. Lightning injuries are the highest during the summer months. However, in some countries such as India and Vietnam, lightning mostly occurs during the rainy season ^5, 6^. Lightning injuries and related deaths mostly affect individuals who work outside or participate in outdoor recreational activities. Worldwide, men are five times more likely than women to be struck by lightning ^3, 7^. The most vulnerable age for lightning injury is estimated to be between 10 and 29 years ^3^.

Lightning injuries cause high mortality and significant long-term morbidity. A previous study reports that in Bangladesh, the incidence of lightning fatalities is 0.9 per 1,000,000 people per year, which is higher than in high-income countries ^6^. In 2016, the country had a lightning event with several strikes, causing 81 deaths, which is particularly high. However, underreporting of lightning strikes is common, as the majority of lightning occurs in rural areas. People used to seek treatment from the local village doctor, pharmacist or traditional healer rather than seeking health care from government facilities, unless the community health provider failed to manage the injuries. Moreover, only a few cases are reported to the police and government hospital records only have information on those who seek treatment. Therefore, studying the epidemiology of lightning injuries in Bangladesh is very important. This study explores the epidemiology of lightning injury, using data from a nationwide survey and newspaper reports on lightning deaths on 13–15 May 2016.

## Methods

The study was a mixed method study using both quantitative and qualitative data. A cross-sectional study was conducted to understand the epidemiology of the lightning injuries in Bangladesh (see below). In addition, we searched two of the most popular Bengali and another three national, English-language newspapers in Bangladesh. Furthermore, lightning news reported in another three international English-language daily newspapers and on three international media websites was retrieved and reviewed ( Table 1). Qualitative data related to lightning injury in Bangladesh were collected to explore the magnitude of lightning injuries in Bangladesh during 13–15 May 2016.

**Table 1.** National and international newspapers and electronic media used in the data search.

Type of newspapers/electronic media	Name of the newspapers/electronic media
National Bengali newspapers	*Prothom Alo*, The *Samakal*
National, English-language newspapers	*The Daily Star*, *The Daily Sun*, *The Independent*
International newspapers	*The Hindu*, *Indian Express*, *The Telegraph*
International electronic media	*National Geographic*, Fox News, Reuters

### Quantitative method

A large cross-sectional study was conducted during January to December 2003 in twelve randomly selected districts in Bangladesh and also in Dhaka Metropolitan City. Multi-stage cluster sampling was employed to select 171,366 households (88,380 in rural areas and 45,183 in urban areas in the twelve districts, and 37,803 in Dhaka Metropolitan City). The current study is part of this larger study. Each district consists of several *upazila*s (subdistricts). From each district, one *upazila* was chosen. The *upazila*s contain smaller units called “union”. A union is the lowest administrative unit, with a population of approximately 20,000. In this study, two unions from each of the *upazila*s were selected. Similarly, in urban settings, the *mohalla* is the lowest unit of the City Corporation. Systemic random sampling was performed of a certain number of households in selected *mohalla*s.

Prior to data collection, 48 trained interviewers had visited the selected households and explained the study objectives and ethical issues. They then conducted the questionnaire survey. As well, 819,429 people of all age groups from 171,336 households in those twelve districts were selected and interviewed using face-to-face interviews.

Persons who were injured by lightning and received treatment or who were unable to perform their usual activities for at least 3 days because of lightning injury were enrolled in the study. We also interviewed the next of kin of people who had died from lightning injuries. About 2.7% of households could not be interviewed because of unavailability of respondents in the households. A total of 166,766 households were included in the study. The methodology has been described elsewhere ^8, 9^.

### Review of the newspapers and electronic media subjected to content analysis

Daily national popular Bangladeshi and English-language electronic newspapers were searched for reports on lightning injuries. Two Bengali and three English-language national newspapers which are widely read in Bangladesh were selected. In addition, we searched international online news sites. Three international English-language newspapers and three purposively selected international online news sites were also included in the search.

As previously mentioned, a high number of lightning events have been reported in Bangladesh for the period 13–15 May 2016. Therefore, we reviewed newspapers to find information on these events. Two researchers collected the relevant information from the selected sources. The next morning, two different researchers sat together and read the news headlines to select relevant articles and eliminate duplicate news. They further read all collected news and then made a further selection pertaining to the study aims. The researchers, who were bilingual, also translated the Bengali news into English.

Only articles relevant to the aims of this study have been included in this study. Each newspaper article constitutes a unit of analysis. Two qualitative researchers conducted content analysis. To determine the overall content and framing of the article, the researchers read, re-read and annotated the news articles by attaching key words to segments of text ^10^.

### Statistical analysis of quantitative data

Standard descriptive statistics using means, standard deviation (SD) and proportions were used to analyse the characteristics of lightning victims. The gender, age, and place of residence of cases of lightning injuries were determined. Cases were categorized into eight age groups. The yearly incidence of lightning injuries was estimated from the occurrence of lightning morbidity in 6 months, multiplied by 2. The reason was that data were collected with a recall period of 6 months. Rates were calculated and 95% confidence intervals (CIs) computed. We estimated the relative risks (RRs) in relation to different age groups, place of residence, and gender. We used cross-tables and EPI-Info 6 software.

### Ethical issues

The current study formed part of a larger study titled “Bangladesh Health and Injury Survey (BHIS)”. The study has received ethics approval from the Ethics Committee of the Institute of Child and Mother Health, Dhaka. Participants were informed about the benefits and objectives of the study. Written consent was obtained from each head of household before proceeding with the interviews. The participants were told that they had the right to withdraw from the study at any time and the study objective was explained to them. Data collectors were trained in ethical issues.

Media news was publicly available. Information from media was anonymously presented without any direct quotation from the media reports. Also the study had not used any personal identification and information related to media reports.

## Results

### Quantitative findings

**Figure 1.**
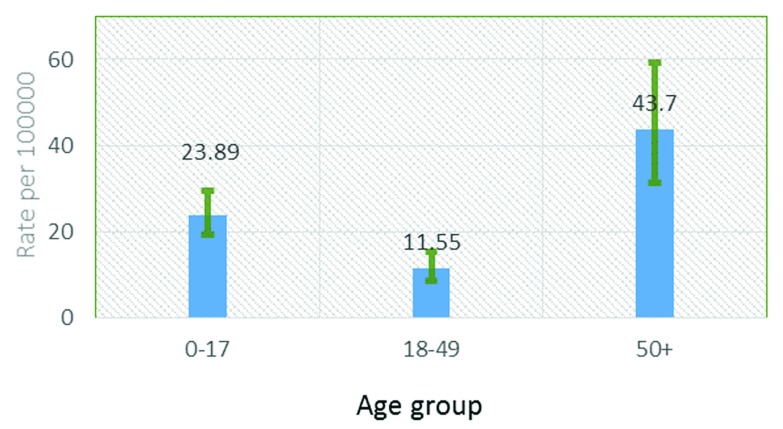
Annual incidence of lightning injuries by age group.

***Incidence.*** A total of 163 people with lightning injuries were identified, 98 males (60.12%) and 65 females (39.8%). Of them, 160 (98.15%) had suffered non-fatal injury and three (1.84%) had died. The annual death rate was 3.661 (95% CI 0.9313–9.964) per 1000 people. The overall incidence of lightning injury was 19.89 per 100,000 people. Males were more vulnerable, with a 1.46 times higher risk of being hit by lightning compared with females (RR 1.46, 95% CI 1.06–1.99). The mean age of the victims was 26.2 (SD±21.83) years (range 2–75 years). Altogether 84 (51.5%) of those struck by lightning were children. The highest incidence of injuries was found in the age group of 50 and above ( Figure 1).

***Magnitude of the injury.*** The majority of victims were of poor socioeconomic status, 86.7% (n=139), with a monthly income of <US$100. Students (31.2%), agricultural workers (17.9%) and housewives (14.5%) were the main victims of lightning injury. Among the victims, 90.80% (n=148) were from rural areas and 9.20% (n=15) from urban areas. People from rural areas were more vulnerable, with an 8.73 times increased risk compared with urban populations (RR 8.73; 95% CI 13–14.86).

About 36% (n=59) of the injuries took place between 6 am and 12 pm, while 43.2% (n=71) occurred between 12 noon and 6 pm, and 18% (n=29) from 6 pm to midnight. A total of 31.7% of victims were outside at work when lightning struck; 24.6% were travelling when they were hit by lightning. Home courtyards were the most common places (65.1%) for lightning strikes, followed by roads and footpaths (26%).

The leg was the most common site of injury, with an incidence of 63.5% (n=97), followed by hand injury in 17.4% of cases (n=27) and abdomen injury in 10.5% (n=16). Among the casualties, 95% (n=155) sought treatment from different level health care providers, with the majority of people (n=134) seeking treatment from the village doctor or traditional healer (83.1%). Only 7.3% (n=8) received treatment at a health facility. Among the injured, 41.8% (n=68) were unable to perform regular activities for 1–6 days while 19.1% (n=31) were unable to do so for ≥1 week. Only 1% (n=2) of the injured reported the incident to the police ( Table 2).

**Table 2.** Lightning victims’ occupation, activity at the time of the lightning strike, time when the injury occurred, place of injury, body part injured, treatment sought, workdays lost due to injury, and treatment providers.

Occupation	%
Student	31.2
Agricultural worker	17.9
Service	2.1
Business	3.1
Housewife	14.5
Other	31.2
**Activity at the time of the strike**	
Work	31.7
Sport (outdoors)	8.3
Leisure/play (indoors)	21.5
Travelling	24.6
Other	12.4
**Time when the injury occurred**	
Midnight – 6 am	2.5
6 am – 12 noon	36.3
12 noon – 6 pm	43.2
6 pm – midnight	18
**Place of injury**	
Home (courtyard)	65.1
Highway/road/footpath	26.0
Agricultural field/farm, excluding home	2.5
Other	6.4
**Body part**	
Head	7.9
Abdomen	10.5
Hand	17.4
Leg	63.5
**Treatment sought**	
Yes	95.0
No	5.0
**Workdays lost**	
<1 week	41.8
1 week – 1 month	16.7
1–3 months	2.4
**Treatment provider**	
Health facilities (clinic or hospital)	7.3
Village doctor	3.3
Homeopathic doctor	2.4
Herbal medicine practitioner	52.6
Traditional healer	29.5

### Findings from media reports

**Figure 2.**
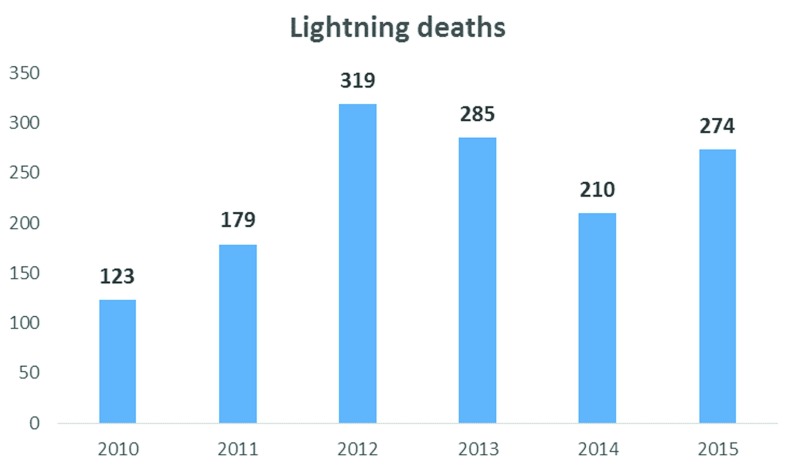
Magnitude of lightning injury from 2010 to 2015, according to the Foundation for Disaster Forum.

Bangladesh has had a high incidence of preventable deaths from lightning for decades. Data on the period 2005–2016 showed that the highest number of deaths in a single day was in May 2016, when lightning killed 81 people in 26 districts, mostly in the rural north and central Bangladesh ^11– 13^. By comparison, lightning deaths between 2005 and 2008 totalled 41. Over the next few years, the number of deaths progressively increased. The English-language Bangladeshi newspaper *Daily Star* reports that from 2010 to 2016 a total of 645 people died in thunderstorms ^14^. Another source, the Foundation for Disaster Forum in Bangladesh, reports 1390 deaths due to lightning for the period 2010–2015 ^15^ ( Figure 2). Other newspapers have reported that an average of 300 people die every year in Bangladesh due to lightning; however, this is underreporting ^11, 16, 17^.

According to the newspaper reports, the youngest person who died from lightning was 13 years old, and the oldest lightning victim was 70 years. In most cases, lightning occurred outdoors in a rural area while the person was performing daily household work or other usual activities. One newspaper reported that 51% of the fatalities were farmers who were working in the fields.

According to the *National Geographic*, lightning storms in Bangladesh occur mostly in May and in the afternoon, when the temperature is high. The fact that the country is densely populated contributes to the high incidence of human lightning strikes. Other sources also mentioned an increase in deforestation, and the felling of tall trees, as a contributing factor. In addition, use of metal objects such as mobiles or structures such as cell phone towers or electrical power distribution towers can result in lightning deaths. It was also mentioned that in rural areas, taller trees usually attract lightning flashes. Internationally, scientists have warned that an increase in lightning storms may happen as part of climate change and global warming. Global warming is causing more water evaporation, increasing cloud formation, the amount of rainfall and the potential for lightning storms ^18– 20^.

After the fatal lightning injury event in May 2016, the Bangladesh government declared lightning a disaster, adding lightning injuries to the country’s list of official types of natural disasters, which includes droughts, floods, cyclones, storm surges and riverbank erosion, and earthquakes ^20, 21^. In 2016 the government pledged to compensate lightning strike victims and/or their families ^12, 22^.

## Discussion

Lightning injury has been identified as one the major causes of weather-related deaths in Bangladesh. In response to the lightning event in 2016, when 81 lives were lost in just 2 days due to lightning, the government of Bangladesh has declared lightning a natural disaster ^21, 23^. The magnitude of the problem has become worse over recent years. According to the current study the annual incidence is 19.89/100,000 population. The majority of victims were males from rural communities, and most injuries were incurred in the afternoon. Labour-intensive agricultural economy, poor infrastructure, illiteracy, and a tropical climate play a role in higher rates of lightning-related deaths and injuries in countries such as South Africa, Malaysia, India and Bangladesh ^19^. For example, one study reports 6.3 deaths/1,000,000 inhabitants in a region mainly populated by the urban poor in Highveld, South Africa ^7^. 

By contrast, a decline in lightning fatalities in recent decades has been reported from developed countries ^24– 27^, reasons for which are: development of medical responses and treatments; education of the public; meteorological warnings; and improved building codes for lightning protection. The latter include housing structures with grounded plumbing, electric conducting materials, improved fire resistance of homes, and lightning rods ^28^.

A previous study reports an annual death rate due to lightning in Bangladesh of 0.9 per 1,000,000 population ^6^. Our study presents the annual death rate as 3.661 (95% CI 0.9313–9.964) per 1,000,000 people. However, these figures are probably underreported because of a poor vital registration system. Lightning deaths are not currently reported in the health system or in the police recording system, which is reliable for public health researchers ^26^. In the United States the number of deaths due to lightning has declined significantly, but the challenge remains to accurately capture the number of deaths ^25^.

We have found that males are most affected by lighting injuries. The majority of victims are from rural communities and were hit in the summertime, in the afternoon. These results correlate with previous studies ^3, 4, 29^. People living in rural communities in Bangladesh have a number of misconceptions including religious myths and superstitions, as well as social stigma attached to lightning injuries ^5, 24, 30, 31^. An initiative has already been taken in an African region to raise awareness of preventive measures against lightning injury among the population to reduce the number of lightning-related deaths and injuries per year ^25^.

## Conclusion

Lightning injuries are important to study in an epidemiological context. In the context of Bangladesh, lightning has become a public health issue that requires urgent action. The country is becoming increasingly urbanized, and has a very high population density. However, rural communities still make up about 70% of the total population. A public lightning awareness programme and the eradication of traditional or religious myths, as well as other preventive measures, such as installing lightning protection systems, can reduce the fatality rate. A multi-stakeholder involvement is required at this stage, including medical doctors, public health professionals, engineers, meteorologists and political leaders, to identify possible and effective solutions for preventing lightning-related deaths. Moreover, it is also important to establish an emergency pre-hospital care system for lightning victims in rural communities, as well as a comprehensive vital registration system that records each death, for future preventive action.

## Data availability

The data referenced by this article are under copyright with the following copyright statement: Copyright: © 2016 Biswas A et al.

Data is stored at the Department of Public Health Sciences and Injury Prevention of CIPRB. Data sharing is subject to the ethical committee’s further permission due to sensitivity and other restrictions. Data can be made available upon detailed request to the corresponding author. The corresponding author will then communicate directly with ethical committee and liaison between the third party willing to avail of the data and the ethical committee.

## Supplementary material

Morbidity questionnaire, mortality questionnaire, and a list of newspaper articles used in this study.
