# Supplementary material for: Lightning Injury is a disaster in Bangladesh? - Exploring its magnitude and public health needs
Source: F1000Res. 2016 Dec 29;5:2931. [Version 1] doi: 10.12688/f1000research.9537.1 (PMC5288675; doi:10.12688/f1000research.9537.1)
Supplement: Supplementary file 1 [file f1000research-5-10274-s0000.tgz › 883e3a7c-c563-471f-a8e8-a32fdb2644b0.zip!/Injury morbidity_questionaire_BHIS.doc]

## Form - 5

*Pattern and Determinants of Child Mortality and Morbidity due to Injury in Bangladesh*


### Date of interview

**Name of the Data Collector:**

**____________________________________**

**Code No.**

**Name of the Supervisor:**

**___________________________________**

# Injury Morbidity Questionnaire

#### Part A : *Information on Injury Morbidity*

1. Intent:

1. Unintentional
2. Intentional self-harm
3. Assault (interpersonal violence)	4. Undetermined
8. Other (specify)______________	9. Unknown

2. Place: Where was the person when s/he was injured?

01. Home

1. Bed room 2. Living room 3. Kitchen

4. Bathroom 5. Yard	6. One room dwelling

8. Other (specify)___________________

02. Educational institute

1. Classroom	2. Play ground	8. Other (specify)___________

03. Highway/street	04. Hostel of the Residential Institution

05. Sports and athletics area

06. Other transport area(Rail station, ferry/launch station, bus stand etc.)

07. Industrial setting/factory/workshop	10. Construction site

11. Agricultural field/ Farm, excluding home

12. Commercial place (market, bazar, office etc.)

13. Water reservoir	88. Others (specify)__________	99. Unknown

3. Date of injury

4. Time of injury (If known, use 24- hour clock)

5. Activity: What was the person doing when s/he was injured?

1. Work	2. Education	3. Sport(outdoor)

4. Leisure/play(indoor)

5. Travelling	8. Other (specify) ______________

9. Unknown

6. Nature of injury

01. Fracture
02. Sprain/Strain or dislocation
03. Cut, bite or open or lacerated wound
04. Amputation
05. Burn	06. Concussion

07. Organs system injury

1. Poisoning	2. Crush injury	3. Blunt trauma

10. Asphyxiation

1. Drowning	2. Burial/suffocation	3. Hanging

4. Smoke/gas inhalation

88. Other (specify) ______________	99. Unknown

7. Part(s) of the body injured

**X	=** fracture

**―	=** amputation

**→ =** Small area

Laceration/ burn

= Large area

Laceration/burn

1. Head	2. Neck	3. Face

4. Chest

5. Abdomen, waist, pelvis

6. Upper extremity
7. Lower extremity
8. Spine

Right

Left

8. Mechanism: How was the person hurt? Or How was the injury inflicted?

01. Traffic injury
02. Assault
03. Fall
04. Stuck/hit by falling object
05. Injury by sharp objects -stab/cut/amputation
06. Gun shot
07. Burn

1. Flames	2. Hot object	3. Hot liquid

4. Chemical	5. Acid	6. Electrical heat

7. Explosive

10. Asphyxiation

1. Drowning
2. Hanging	3.Choking

4. Inhalation of gas/smoke

11. Accidental Poisoning

12. Machine/tool injury (laceration, crush, amputation)

13. Electrocution
    1. Lightening
    2. Electricity

14. Bite of animal or insect ()

88.	Other (specify) _____________________

99. Unknown

9. Treatment received for the injury:

1. Yes	2. No (Ask question 18)

10. Treatment provider:

01.Government hospital/health centre/clinic

02. Private hospital/health centre/clinic

03. Private registered doctor

04. Medical Assistant (SACMO)

05. Health Assistant/Family Welfare Visitor/Family Welfare Assistant

06. NGO Service provider

07. Medicine shopkeeper/Village doctors

10. Homeopathic practitioner

11. Herbal medicine practitioner

12. Trained TBA

13. Traditional healer/religious leader

14. Relative/friends

88. Others (specify) __________________

11. If the injured person was taken to hospital/health centre/clinic, then

– type of hospital

1. Specialized Hospital (Medical College Hospital, Orthopaedic Hospital, Post graduate Hospital etc.)
2. District Hospital
3. Upazila Hospital
4. Private Clinic
5. NGO Clinic
8. Others (specify) __________________

12. Transportation to hospital/other treatment provider

1. Ambulance
2. Other motorized vehicle (bus, jeep, car and three wheeler)
3. Non-motorized vehicle (rickshaw, rickshaw van, cart etc.)
4. Boat
5. Ferry/launch/ Steamer
6. Treatment provider visited the injured person at home
8. Others (specify) ______________________

13. Time elapsed to reach the hospital/treatment provider after injury

1. With in 30 minutes
2. 30 min to 1 hour
3. 1 hour to 6 hours
4. 6 hours to 24 hours
5. 24 + hours

9. Unknown

14. Was surgery/operation done?

1. Yes	2. No

15. If hospitalized, duration of hospital stay (days)

16. Out come of treatment:

1. Recovered	2. Improved

3. Referred to other hospital

4. No improvement

17. Total treatment cost (BDT)

18. If disability, what was that?

1. Loss of eye sight	2. Loss of hearing

3. Loss of mobility	4. Loss of activity with hands

5. Loss of speech	6. Loss of intellect

8. Others (specify) ____________

9. No permanent disability

19. If the above answer is “1 - 4”, then

1. Loss of eye sight	1. Unilateral	2. Bilateral

2. Loss of hearing	1. Unilateral	2. Bilateral

3. Loss of mobility	1. Unilateral	2. Bilateral

4. Loss of activity with hands	1. Unilateral	2. Bilateral

20. How many days this disability last?

1. < 1 week	2. > 1 week but < 1 month

3. > 1 month but < 3 month	4. > 3 month but not permanent

5. Permanent

21. If temporary disability

*(After discharge from hospital or completion of treatment at home to normal activity)*

21.1. Number of days required help for activities of daily life (ADLs)

21.2. Number of days of school unattended

*(if not applicable fill in the boxes with 999)*

21.3. Number of days lost from work

*(if not applicable fill in the boxes with 999)*

22. Injury event reported to upazila police station

1. Yes	2. No

23. Is the person a significant source of income for the family?

1. Main income earner or labour

2. Major but not main

3. Minor

4. None

5. Unable to answer

24. What has been economic impact on family due to disability of the person?

1. Major but temporary

2. Major but permanent

3. Minor, temporary

4. Minor permanent

5. None

25. In the month before the injuy was the person sick or ill that required medical attention or caused them to miss 1 day or more of school or work?

1. Yes	2. No

26. Had the person been injured by –

(1. Yes	2. No)

| 1. Intentional self-harm (Suicide) - *Collect detailed information using M-1* |  |
| --- | --- |
| 2. Traffic injury - *Collect detailed information using M – 2* |  |
| 3. Assault - *Collect detailed information using M – 3* |  |
| 4. Fall *Collect detailed information using M – 4* |  |
| 5. Stuck/hit by falling object - *Collect detailed information using M – 5* |  |
| 6. Injury by sharp objects - *Collect detailed information using M –6* |  |
| 7. Gun shot |  |
| 8. Burn – flames, hot object, hot liquid, chemical, acid, electrical heat, explosive - *Collect detailed information using M – 7* |  |
| 9. Asphyxiation – drowning - *Collect detailed information using M – 8*   hanging, chocking, inhalation of gas/smoke |  |
| 10. Accidental Poisoning - *Collect detailed information using M – 9* |  |
| 11. Machine/tool injury - *Collect detailed information using M – 10* |  |
| 12. Electrocution - lightening, electricity - *Collect detailed information using M – 11* |  |
| 13. Bite of animal or insect (dog, cat or snake bite, bee or wasp sting) - *Collect detailed information using M –12* |  |

***M – 11 Information on Electrocution***

1. What was the source?

**Form 5, M-11, 12**

1. Lightning

2. Electric using in home (Ask question 2)

3. Electric using in outside home (Ask question 3)

4. Electric using in factory

5. Electric using in building other than house or factory

2. If home, where occur?

1. Kitchen	2. Living area	3. Bed room	4. Bathroom

8. Other (specify) __________

3. If outside home, Where?

1. Traveling	2. Working in field	3. Working in factory/Office

4. School, institute	8. Other (specify) ___________________

***M – 12 Information on Animal bite and insect bite/sting***

***Animal bite***

1. Name of animal

01. Pet dog	02. Stray dog	03. Pet cat	04. Stray cat

05. Snake	06. Fox	07. Cow	10. Ox

11. Goat/sheep 12. Buffalo

88. Other (specify) ______________________

2. Type of injury

1. Bite	2. Kick	3. Step on

4. Horn/other butting/impale

3. Why bitten/butted/impaled?

1. Playing/disturbing	2. Feeding	3. Using for work

4. Unprovoked, no prior involvement	5. Rabid animal

8. Other (specify)__________________

***Insect bite/sting***

4. What insect?

1. Bee/wasp	2. Scorpion	3. Spider	4. Caterpillar

8. Other (specify)_________________
