# Supplementary material for: Lightning Injury is a disaster in Bangladesh? - Exploring its magnitude and public health needs
Source: F1000Res. 2016 Dec 29;5:2931. [Version 1] doi: 10.12688/f1000research.9537.1 (PMC5288675; doi:10.12688/f1000research.9537.1)
Supplement: Supplementary file 1 [file f1000research-5-10274-s0000.tgz › 883e3a7c-c563-471f-a8e8-a32fdb2644b0.zip!/Injury mortality_questionaire_BHIS.doc]

## Form - 4

*Pattern and Determinants of Child Mortality and Morbidity due to*

*Injury in Bangladesh*


**Name of the Data Collector:**

**_____________________________________**

### Date of interview

**Name of the Supervisor:**

**___________________________________**

**Code No.**

***Injury Mortality*** ***Questionnaire***

#### Part A *:* Information on Injury Mortality

1. Intent:

1. Unintentional

2. Intentional self-harm

3. Assault (interpersonal violence) 4. Undetermined

8. Other (specify)______________ 9. Unknown

2. Place: Where was the person when s/he was injured?

01. Home

1. Bed room 2. Living room 3. Kitchen

4. Bathroom 5. Yard	6. One room dwelling

7. Other (specify)___________________

02. Educational institute

1. Classroom	2. Play ground 8. Other (specify)___________

03. Highway/street	04. Hostel of the Residential Institution

05. Sports and athletics area

06. Other transport area (rail station, ferry/launch station/bus station)

07. Industrial setting/factory/workshop	10. Construction site

11. Agricultural field/ Farm, excluding home

12. Commercial place (market, bazar, office etc.)

13. Water reservouir (pond, ditch, canal, river etc.)

88. Others (specify)__________	99. Unknown

3. Place of death

1. Spot of injujry	2. Hospital 3.Home 4. On the way to home
5. On the way to hospital 8.Others (specify) _____________________

4. Date of injury

5. Time of injury (If known, use 24- hour clock)

6. Time of death (If known; use 24-hour clock)

7.	Activity: What was the person doing when s/he was injured?

1. Work	2. Education	3. Sport (outdoor)

4. Leisure/play (indoor)	5. Travelling

8. Other (specify) ______________	9. Unknown

8. Nature of injury

01. Fracture
02. Sprain/Strain or dislocation
03. Cut, bite or open wound, lacerated wound
04. Amputation
05. Burn	06. Concussion

07. Organs system injury

1. Poisoning	2. Crush injury	3. Blunt trauma

08. Asphyxiation

1. Drowning	2. Burial/suffocation	3. Hanging

4. Smoke/gas inhalation

88. Other (specify) ______________	99. Unknown

9. Part(s) of the body injured

**X	=** fracture

**―	=** amputation

**→ =** Small area

Laceration/ burn

= Large area

Laceration/burn

1. Head	2. Neck	3. Face

4. Chest

5. Abdomen, waist, pelvis

6. Upper extremity
7. Lower extremity
8. Spine

Rt

Lt

10. Mechanism: How was the person hurt? Or How was the injury inflicted?

01. Traffic injury
02. Assault
03. Fall
04. Stuck/hit by falling object
05. Injury by sharp objects -stab/cut/amputation
06. Gun shot
07. Burn

1. Flames	2. Hot object	3. Hot liquid

4. Chemical	5. Acid	6. Electrical heat

7. Explosive

10. Asphyxiation

1. Drowning
2. Hanging	3.Choking

4. Inhalation of gas/smoke

11. Accidental Poisoning

12. Machine/tool injury (laceration, crush, amputation)

13. Electrocution
    1. Lightening
    2. Electricity
14. Bite of animal or insects

88.	Other (specify) _____________________

99. Unknown

11. Treatment received for the injury:

1. Yes	2. No (Ask question 19)

12. Treatment provider:

01. Government hospital/health centre/clinic

02. Private hospital/health centre/clinic

03. Private registered doctor

04. Medical Assistant (SACMO)

05. Health Assistant/Family Welfare Visitor/Family Welfare Assistant

06. NGO Service provider

07. Medicine shopkeeper/Village doctors

10. Homeopathic practitioner

11. Herbal medicine practitioner

12. Trained TBA

13. Traditional healer/religious leader

14. Relative/friends

88. Others (specify) __________________

13. If the injured person was taken to hospital/health centre/clinic,	type of hospital -

1. Specialized Hospital (Medical College Hospital, Orthopaedic Hospital, Post graduate Hospital etc.)
2. District Hospital
3. Upazila Hospital
4. Private Clinic
5. NGO Clinic
8. Others (specify) __________________

14. Transportation to hospital/other treatment provider

1. Ambulance
2. Other motorized vehicle (bus, jeep, car and three wheeler)
3. Non-motorized vehicle (rickshaw, rickshaw van, cart etc.)
4. Boat
5. Ferry/launch/ Steamer
6. Treatment provider visited the injured person at home
8. Others (specify) ______________________ 9. Not known

15. Time elapsed to reach the hospital after injury/ treatment provider after injury

1. With in 30 minutes
2. 30 min to 1 hour
3. 1 hour to 6 hours
4. 6 hours to 24 hours
5. 24 + hours

9. Not known

16. Was surgery/operation done?

1. Yes	2. No

17. Duration of Hospital stay (days)

18. Total treatment cost (BDT)

19. Injury event reported to upazila police station

1. Yes	2. No

20. Post-mortem conducted

1. Yes	2. No

21. Was the person a significant source of income for the family?

1. Main income earner or labour

2. Major but not main

3. Minor

4. None

9. Unable to answer

22. What has been economic impact on family of the death

1. Major but temporary

2. Major but permanent

3. Minor, temporary

4. Minor permanent

5. None

23. In the month before death was the person sick or ill that required medical attention or caused them to miss 1 day or more of school or work?

1. Yes	2. No

24. Had the person been injured by –

(1. Yes	2. No)

| 1. Intentional self-harm (Suicide) - *Collect detailed information using M-1* |  |
| --- | --- |
| 2. Transport injury - *Collect detailed information using M – 2* |  |
| 3. Assault (interpersonal violence)- *Collect detailed information using M – 3* |  |
| 4. Fall - *Collect detailed information using M – 4* |  |
| 5. Stuck/hit by falling object - *Collect detailed information using M – 5* |  |
| 6. Injury by sharp objects - *Collect detailed information using M –6* |  |
| 7. Gun shot |  |
| 8. Burn – flames, hot object, hot liquid, chemical, acid, electrical heat, explosive - *Collect detailed information using M – 7* |  |
| 9. Asphyxiation – drowning - *Collect detailed information using M – 8*   hanging, chocking, inhalation of gas/smoke |  |
| 10. Accidental Poisoning - *Collect detailed information using M – 9* |  |
| 11. Machine/tool injury - *Collect detailed information using M – 10* |  |
| 12. Electrocution - lightening, electricity - *Collect detailed information using M – 11* |  |
| 13. Bite of animal or insect – dog, cat or snake bite, bee or wasp sting - *Collect detailed information using M –12* |  |

25. Had the person ever been diagnosed with –

(1. Yes	2.No)

1. Epilepsy

2. Mental retardation

3. Mental illness

**Part B: *Mechanism of injury***

***M – 11 Information on Electrocution***

**Form 4, M-11,12**

1. What was the source?

1. Lightning

2. Electric using in home (Ask question 2)

3. Electric using in outside home (Ask question 3)

4. Electric using in factory

5. Electric using in building other than house or factory

2. If home, where occur?

1. Kitchen	2. Living area	3. Bed room	4. Bathroom

8. Other (specify) __________

3. If outside home, Where?

1. Traveling	2. Working in field	3. Working in factory/Office

4. School, institute	8. Other (specify) ___________________

***M – 12 Information on Animal bite and insect bite/sting***

***Animal bite***

1. Name of animal

01. Pet dog	02. Stray dog	03. Pet cat	04. Stray cat

05. Snake	06. Fox	07. Cow	10. Ox

11. Goat/sheep 12. Buffalo

88. Other (specify) ______________________

2. Type of injury

1. Bite	2. Kick	3. Step on

4. Horn/other butting/impale

3. Why bitten/butted/impaled?

1. Playing/disturbing	2. Feeding	3. Using for work

4. Unprovoked, no prior involvement	5. Rabid animal

8. Other (specify)__________________

***Insect bite/sting***

4. What insect?

1. Bee/wasp	2. Scorpion	3. Spider	4. Caterpillar

8. Other (specify)_________________
