# Supplementary material for: Lightning Injury is a disaster in Bangladesh? - Exploring its magnitude and public health needs
Source: F1000Res. 2016 Dec 29;5:2931. [Version 1] doi: 10.12688/f1000research.9537.1 (PMC5288675; doi:10.12688/f1000research.9537.1)
Supplement: Supplementary file 1 [file f1000research-5-10274-s0000.tgz › 883e3a7c-c563-471f-a8e8-a32fdb2644b0.zip!/Table_Newspaper_media report on lightning injury.doc]

| **Title of the newspaper / media** | **Newspaper/ media** | **URL** | **Date of accessed** |
| --- | --- | --- | --- |
| Lightning kills 81 people in two days in Bangladesh. | The Indain Express | http://indianexpress.com/article/world/world-news/lightning-kills-81-people-in-two-days-in-bangladesh-2805418/ | 17 May 2016 |
| Lightning claims 81 lives across Bangladesh in two days. | The Daily Samakal | http://www.samakal.net/2016/05/17/5769 | 17 May 2016 |
| Lightning strikes killed 81 people across Bangladesh during recent thunderstorms , says government | bdnews24.com | http://bdnews24.com/bangladesh/2016/05/17/lightning-strikes-killed-81-people-across-bangladesh-during-recent-thunderstorms-says-government | 17 May 2016 |
| Lightning takes 17 more lives in 11 districts. | The Daily Star | <http://www.thedailystar.net/c>  untry/2-killed-lightning-ctg  gaibandha-1223248 | 21 May 2016 |
| Awareness can reduce death rate from lightning. | The Daily sun | http://www.daily-sun.com/printversion/details/134718/Awareness-can-reduce-death-rate-from-lightning | 8 May 2016 |
| Bangladesh lightning death toll rises to 35. 2016 | The Indain Express | http://indianexpress.com/article/world/world-news/bangladesh-lightning-death-toll-rises-to-35-2798108/ | 13 May 2016 |
| Bangladesh lightning death toll rises to 35 | The Hindu | <http://www.thehindu.com/news/international/bangladesh-lightning-death-toll-rises-to-35/article8593822.ece> | 13 May 2016 |
| More than 60 killed by lightning in Bangladesh in two days. | FOXNEWS World | http://www.foxnews.com/world/2016/05/14/up-to-64-killed-by-lightning-in-bangladesh-reports-say.html | 27 May 2016 |
| Death by Lightning a Danger in Developing Countries | National Geographic | [http://news.nationalgeogra](http://news.nationalgeogra/)  hic.com/news/2013/11/131  02-lightning-deaths  developing-countriesstorms/ | 23 May 2016 |
| Bangladesh declares lightning strikes a disaster as deaths surge. | REUTERS | <http://in.reuters.com/article/bangladesh-lightning-disaster-idINKCN0Z81U4> | 02 June 2016 |
| Lightning kills at least 93 as  monsoon sweeps India. | The Telegraph | http://www.telegraph.co.uk/news/2016/06/22/lightning-kills-at-least-93-as-monsoon-sweeps-india/ | 07 June 2016 |
| Lightning now a disaster : Ministry | The Indepdendent | http://www.theindependentbd.com/post/44195 | 15 May 2016 |
| Lightning : The New Natural Disaster. | The Daily Star | http://www.thedailystar.net/star-weekend/lightning-the-new-natural-disaster-1291867 | 24 June 2016 |
