# Supplementary material for: Lightning Injury is a disaster in Bangladesh? - Exploring its magnitude and public health needs
Source: F1000Res. 2016 Dec 29;5:2931. [Version 1] doi: 10.12688/f1000research.9537.1 (PMC5288675; doi:10.12688/f1000research.9537.1)
Supplement: Peer review history

# Peer review: Referee response for version 1

**DOI:** 10.5256/f1000research.10274.r19744

**Published:** 2017-02-01

**Authors:** Mithila Faruque

**Affiliations:** 1 Department of Noncommunicable Diseases, Bangladesh University of Health Sciences (BUHS), Dhaka, Bangladesh

I have gone through the article and I think this is a public health concern and I would like to thank the authors for depicting the timely problem in front of the readers. Definitely it is a good initiative. I only have the following comments to clarify by the authors:

**Title:**

 The title would be more appropriate if make it in one sentence and more simple.

**Methods:**

1. Which area you have taken as urban and rural in the study should be specifically explained. Dhaka Metropolitan City and Upazila cannot be the same as urban area.
2. Why has Dhaka been selected separately out of 12 random district selections?
3. What was your denominator in the calculation of incidence rate over 6 months?

**Discussion:**

1. If you could compare the results (like study subjects affected more and the lightning circumstances) with other studies, then it would add more value to the study.
2. ‘People living in rural communities in Bangladesh have a number of misconceptions including religious myths and superstitions, as well as social stigma attached to lightning injuries’ – why it came in the discussion? Did you get this type of information from your study?

**Conclusion:**

 Conclusion should contain what you have actually obtained in the study. The others may be a part of the recommendation.

I have read this submission. I believe that I have an appropriate level of expertise to confirm that it is of an acceptable scientific standard.

# Peer review: Referee response for version 1

**DOI:** 10.5256/f1000research.10274.r19557

**Published:** 2017-01-30

**Authors:** Kazi Md Noor-ul Ferdous, Kh. Ahasanul Kabir, Ashfaq Nabi

**Affiliations:** 1 Division of Pediatric Surgery, Dhaka Shishu (Children) Hospital, Dhaka, Bangladesh; 2 Dhaka Shishu (Children) Hospital, Dhaka, Bangladesh; 3 Bangladesh Institute of Child Health, Dhaka, Bangladesh

In the above article the authors have conducted an important but neglected public health issues in Bangladesh. 

 The study is part of national injury survey which is country representative data. They clearly surface the magnitude of the problem and factors associated with this. The authors also matched the findings with newspaper findings of 2016.

 The study is well designed, their title is also clear and specific. The objectives matched with the title and results. Conclusion has been directed about way forward, however, this could be interesting if the authors could discuss on how different stakeholders can involve in the process and work together in reduction of lightning injury and deaths in Bangladesh.

 You may accept the paper to be indexed.

We have read this submission. We believe that we have an appropriate level of expertise to confirm that it is of an acceptable scientific standard.

# Peer review: Referee response for version 1

**DOI:** 10.5256/f1000research.10274.r18837

**Published:** 2017-01-24

**Authors:** Aziz Rahman

**Affiliations:** 1 Austin Health Clinical School of Nursing, La Trobe University, Melbourne, Australia

The authors have focused on an important area of public health research from the perspective of Bangladesh. Due to scarcity of data availability, it is important to publish more evidence based research. The findings of the study are interesting and would shed lights for the future awareness programs and possible policy changes. The following issues need to be addressed for my approval:

1. The title needs to be amended to make it correct grammatically.
2. The article, specifically the abstract needs to be revised for grammatical and language errors.
3. Introduction: Needs to have more discussion on data availability from Bangladesh or neighboring countries.
4. Results: The numbers presented under the incidence are confusing. The authors mentioned that 51.5% were children, who were stuck by lightning, whereas the next line says that the highest incidence was among >50 years old. The authors should consider presenting more inferential analyses (only gender and residence location are presented)
5. Discussion: There should be a comprehensive discussion on the ways forward, how to prevent such incidence mentioning the steps taken in other neighboring countries.

I have read this submission. I believe that I have an appropriate level of expertise to confirm that it is of an acceptable scientific standard, however I have significant reservations, as outlined above.

# Peer review: Referee response for version 1

**DOI:** 10.5256/f1000research.10274.r19402

**Published:** 2017-01-17

**Authors:** Mohammad Delwer Hossain Hawlader

**Affiliations:** 1 Department of Public Health, School of Health and Life Sciences, North South University, Dhaka, Bangladesh

The authors have described one of the most important public health needs for further action. Paper is well structured and written well too. Although the findings is from Bangladesh health and injury survey which conducted in 2003, therefore the epidemiology and factors may be modified in last 10 years. Since, there is no alternative data to compare, this paper is important for the decision makers, at the policy level.

 English of the manuscript is understandable. I would recommend to accept and approve the article to be indexed.

I have read this submission. I believe that I have an appropriate level of expertise to confirm that it is of an acceptable scientific standard.
